# An animal study on the effectiveness of platelet-rich plasma as a direct pulp capping agent

**DOI:** 10.1038/s41598-024-54162-1

**Published:** 2024-02-14

**Authors:** Dina M. Elkady, Yara R. Helaly, Hala W. El Fayoumy, Huda O. AbuBakr, Aya M. Yassin, Naglaa A. AbdElkader, Dina B. E. Farag, Possy M. Abd El Aziz, Antonio Scarano, Ahmad G. A. Khater

**Affiliations:** 1 Conservative Dentistry Department, Faculty of Dentistry, Cairo University, Giza, Egypt; 2 Oral and Maxillofacial Radiology Department, Faculty of Dentistry, Cairo University, Giza, Egypt; 3 Department of Biochemistry and Molecular Biology, Faculty of Veterinary Medicine, Cairo University, Giza, Egypt; 4 Department of Surgery, Anesthesiology, and Radiology, Faculty of Veterinary Medicine, Cairo University, Giza, Egypt; 5 Oral Biology Department, Faculty of Dentistry, Cairo University, Giza, Egypt; 6 Department of Innovative Technologies in Medicine and Dentistry, University of Chieti–Pescara, Chieti, Italy; 7 Faculty of Oral and Dental Medicine, Egyptian Russian University, Badr City, Cairo Egypt; 8 Health Affairs Directorate, Egyptian Ministry of Health and Population, Banisuif, Egypt

**Keywords:** Carious pulp exposure, Dentinogenic biomarkers, Mineral trioxide aggregate, Platelet rich plasma, Reversible pulpitis, Vital pulp therapy, Preclinical research, Translational research, Oral diseases

## Abstract

Direct pulp capping (DPC) is a conservative approach for preserving tooth vitality without requiring more invasive procedures by enhancing pulp healing and mineralized tissue barrier formation. We investigated the effectiveness of Platelet Rich Plasma (PRP) vs. Mineral Trioxide Aggregate (MTA) as a DPC agent. Forty-two teeth from three mongrel dogs were divided into two equal groups. After three months, the animals were sacrificed to evaluate teeth radiographically using cone-beam computerized tomography, histopathologically, and real-time PCR for dentin sialophosphoprotein (DSPP), matrix extracellular phosphoglycoprotein (MEPE), and nestin (NES) mRNA expression. Radiographically, hard tissue formation was evident in both groups without significant differences (*p* = 0.440). Histopathologic findings confirmed the dentin bridge formation in both groups; however, such mineralized tissues were homogenous without cellular inclusions in the PRP group, while was osteodentin type in the MTA group. There was no significant difference in dentin bridge thickness between the PRP-capped and MTA-capped teeth (*p* = 0.732). The PRP group had significantly higher DSPP, MEPE, and NES mRNA gene expression than the MTA group (*p* < 0.05). In conclusion, PRP enables mineralized tissue formation following DPC similar to MTA, and could generate better cellular dentinogenic responses and restore dentin with homogenous architecture than MTA, making PRP a promising alternative DPC agent.

## Introduction

Restorative dentistry has undergone a paradigm shift in recent years, becoming more conservative by developing less-invasive approaches that concentrate on caries pathogenesis and tissue healing capacities to maintain tooth vitality as much as possible^1^. Such initiatives attempt to preserve pulp vitality since it is critical for protecting tooth structure and preserving physiological functions, as it is responsible for tooth viability, pulp nutrition and innervation, and immune response mechanisms^2^. As a result, vital pulp therapy (VPT) seeks to preserve the pulp tissues' vitality and functionality from potential accidents that may lead to the exposure of the vital pulp (dental caries, restorative procedures, iatrogenic etiology, and dental trauma)^3,4^. The VPT is divided into pulp capping (direct and indirect) and pulpotomy (miniature, partial, and complete) based on case selection and diagnosis, exposure site, root maturation, coronal restoration state, and treatment strategy^5^. Direct pulp capping (DPC) is an effective and conservative procedure among these restorative techniques^6^, which involves placing a biomaterial directly over the exposed pulp after caries removal or trauma to maintain the pulp vitality by covering the pulp exposure, preventing bacterial leakage, and promoting dentin bridge formation^7,8^.

Most DPC materials cause superficial necrosis after direct application over exposed pulp tissues^9^; they induce mineralization due to their antimicrobial properties, enabling the underlying pulp cells to heal and regenerate^10^. However, eliminating bacterial organisms does not directly influence reparative dentin formation since these effects are indirect; hence, the evidence on the role of calcium ions and antimicrobial properties of DPC materials in reparative dentin formation remains to be determined^11^. As such, many materials have been used for DPC, yet there is no universal consensus about the best material; the choice usually depends on the clinician’s preference^12^. The optimal DPC material should be easy to apply, adherent, antibacterial, have excellent sealing, insoluble, biocompatible, bioactive, promote mineralization, radiopaque, and not discolor tooth^13^; still, such an ideal material does not exist since each material has limitations.

Mineral trioxide aggregate (MTA) has acquired substantial recognition due to its ability to improve dentin-pulp complex wound healing^12,14,15^, as well as its superior biocompatibility, high sealing capacity, suppression of bacterial invasion, and low solubility^16^. Also, MTA has beneficial physiochemical features that stimulate reparative dentin production, minimize pulp inflammation and necrosis, and solubilize bioactive proteins, improving tooth healing^17–19^. As a result, the clinical effectiveness of MTA in reducing pulp inflammatory response, forming a reparative dentin bridge, and boosting success rate has been well-documented in the literature, making MTA a reliable treatment for exposed pulp and DPC with a predictable outcome^3^. On the other side, MTA has many limitations, including a high cost, difficult application, tooth discoloration, and a long setting time^20^; yet, it remains the standard for pulp capping material derived from hydraulic calcium cement due to the most available and long-term studies on it^21^.

Platelet-rich plasma (PRP) is widely used as a bioactive scaffold in tissue engineering and cell-based therapies^22^. It is made from autologous plasma with concentrated platelets containing more than 300 biologically active molecules, which are released from platelet alpha and dense granules during activation and then control tissue regeneration^23,24^. As a result, the use of PRP is constantly growing, particularly in regenerative dentistry, which includes regenerative endodontics, periodontics, and oral and maxillofacial surgery^25^. PRP has been considered a promising pulp-capping agent due to its superior tissue compatibility and antibacterial properties^26^ and its ability in the mineralization, proliferation, and recruitment of mesenchymal stem cells in the pulp^27^. As such, using PRP for DPC will act as a biomaterial to deliver vital growth factors and cytokines from platelet granules to the targeted location, encouraging dentin-like tissue regeneration^25,28^. Also, PRP is an inexpensive, cost-effective, safe, and aseptic technique since it can be quickly generated from the patient's blood^2^.

Still, the effectiveness of PRP as a DPC agent requires further investigation since most of the current evidence is inconclusive^29^ since basic research was based on histological evaluation, whereas clinical research was mainly focused on radiographic assessment of reparative dentin. In order to address this knowledge gap, this research aimed to correlate the histological evaluation with radiographic assessment and augment these data with gene expression analysis of dentinogenic biomarkers to evaluate the underlying molecular mechanism of PRP's biological activity and regenerative capacity in DPC compared to MTA (gold standard). Therefore, we designed this study to investigate the null hypothesis that no radiographic, histopathologic, and dentinogenic gene expression differences exist between PRP and MTA as DPC agents.

## Methods

### Study design

This study followed the ARRIVE guidelines 2.0 for reporting animal research^30,31^.

### Inclusion criteria

In this study, three healthy adult male mongrel dogs purchased from the Al-Fahad Trading Company of Animals (Abu-Rawash, Giza, Egypt) weighing 25–30 kg and aged 1–2 years were housed in separate kennels under typical ambient conditions (23 °C, 55% humidity, and a 12-h light/dark cycle) with twice-daily maintenance meals and free access to water.

The study followed a split-mouth design involving 14 teeth from each dog: the three incisors (first, second, and third) and canine from each maxillary quadrant, and the two incisors (second and third) and canine from each mandibular quadrant. As such, a total of 42 teeth from both arches were randomly assigned into two groups based on the pulp capping material:

- Experimental group (PRP-capped): 21 teeth from all quadrants (i.e., seven teeth from each dog, including four maxillary and three mandibular teeth).
- Control group (MTA-capped): 21 teeth from all quadrants (i.e., seven teeth from each dog, including four maxillary and three mandibular teeth).

### Sample size

We estimated our sample to represent an alpha level of 0.05, a beta level of 0.2 (80% power), and an effect size of 1.18 from previous research^29^, resulting in a sample size of 42 teeth. Sample size calculation was performed using G*power version 3.1.9.7^32^.

### Randomization and blinding

Based on the DPC material applied, two parallel groups were established: PRP and MTA, and the randomization of these materials for the prepared cavities were as follows: seven identical opaque sealed envelopes numbered one to seven were made, and three operators were asked to choose two envelopes. If the number inside the envelope was even, the teeth were directly pulp capped in the right quadrant with PRP and MTA in the left quadrant; in contrast, PRP was applied to the left side and MTA to the right side if the number was odd. Given the nature of the intervention and the apparent difference between the two materials, it was impossible to make operators (D.M.E and P.M.A) unaware of the material used; however, the outcomes assessors remained blinded about the groups.

### Experimental procedures

#### Autologous PRP preparation

We collected whole-blood samples from the dogs' jugular veins using sodium citrate tubes, which were prepared using the double-spin method and activated with CaCl_2_ shortly before usage, as described by Farghali et al^33^. Briefly, 9 ml of whole blood was collected on 1 ml of 3.8% sodium citrate solution-containing tubes, which were then subjected to soft spin at 250xg for 10 minutes. The resulting upper and middle layers were collected for a hard spin at 2000xg for 10 minutes, while the upper 2/3 portion was discarded as a poor platelet plasma portion, yielding 1.5 ml PRP. PRP was activated with 20Mm CaCl_2_ and then incubated at 37 °C for 1 hour.

#### Dental procedures

All dental procedures were carried out under general anesthesia. Each dog was pre-medicated with atropine sulfate (Atropine; ADWIA Company, Cairo, Egypt) at a subcutaneous dose of 0.1mg/kg and xylazine HCl (Xyla-Ject; ADWIA Company, Cairo, Egypt) at an intravenous dose of 1mg/kg. General anesthesia was induced by a 10 mg/kg intravenous ketamine HCl injection (Ketamine; EPICO, Cairo, Egypt) and sustained with a 2.5% intravenous thiopental sodium solution injection (Thiopental sodium; EPICO, Cairo, Egypt) at 25 mg/kg^34^.

Following general anesthesia, teeth surfaces were cleansed with saline irrigation (El Nasr Pharmaceutical Chemical, Abu Zaabal, Egypt), and the mouth was swabbed with 0.2% chlorhexidine digluconate (Corsodyl 0.2% mouthwash; GlaxoSmithKline Consumer Healthcare, Great West Road, Brentford, Middlesex, England)^34^; then, the jaws were separated using a modified plastic syringe by cutting its upper head^35^.

After rubber dam application, we prepared class V cavities at the neck third of the labial surfaces of the selected teeth parallel to the cementoenamel junction (CEJ) using low-speed round bur under copious irrigation. Such cavities were 2.5 mm wide, 3 mm long, and 1.5–2 mm deep. A new size-2 round bur was used to expose the tooth's pulp, and the cavity was immediately washed with saline after exposure.

In the experimental group, pre-prepared PRP was directly injected on the exposure site with a sterile plastic syringe, and the cavity was covered with an adsorbent membrane to remove excess PRP. In the control group, MTA (ProRoot; Dentsply, Tulsa, OK, USA) was mixed according to manufacturer instructions and applied to the cavity with a small ball burnisher. Finally, all cavities were filled with glass ionomer restoration (EQUIA Forte, GC America).

#### Animals euthanasia and tissue preparation

After three months, the animals were sacrificed via rapid intravenous administration of an overdose of euthanasia solution containing 20% pentobarbitone sodium (20% Euthanasia Injection; Arnolds, UK). The dogs' maxilla and mandibles were immediately dissected free, and teeth were separated from the jaws in blocks, containing the teeth with their surrounding bone. Then, the teeth were extracted from such blocks with a sharp saw, and bone remnants were removed with a sharp stone. Such extracted teeth were immediately cleansed with sterile phosphate-buffered saline and placed on ice, and the subsequent procedures were performed sterilely by transferring the teeth into a laminar flow tissue culture hood for molecular analysis.

#### Pulp extirpation

Afterward, we used discs to cut the apical part of the teeth and cautiously extracted pulp tissues apically with sterile endodontic instruments to preserve the coronal part and the formed reparative dentin. Pulp tissues were immediately frozen in liquid nitrogen and stored at − 80 °C until usage, and the teeth were stored in 10% neutral buffered formalin for radiographic and histological evaluation.

### Outcome measures

#### Radiographic evaluation

Radiographic evaluation was conducted using CBCT Planmeca ProMax 3D Mid (Asentajankatu, Helsinki, Finland) with exposure parameters of 90 kV, 11 mA, 15 s, and 4 × 5 cm field of view with a voxel size of 75 µm. Each tooth was scanned separately to reduce artifacts in CBCT images, then, a serial profile of the corrected sagittal images of the examined tooth was selected for compatibility with histological sections of the dentin bridge, allowing the presence or absence of dentin bridges to be confirmed^36^. Such corrected sagittal images with 0.1 mm thickness were used to detect the presence of hard tissue formed over the exposed pulp, and each tooth was assigned a score in an Excel sheet. Score (1) denotes there is evidence of hard tissue formation (i.e., dentin bridge radiographically observed), while score (0) denotes no evidence of hard tissue formation (i.e., no dentin bridge radiographically observed), and score (000) denotes no restoration found (i.e., restoration was lost during tooth extraction)^37^. Two blinded authors (Y.R.H and H.W.EF) independently assessed the CBCT images using the Planmeca Romexis Viewer Launcher software (version 5.3.3.5 R).

#### Histopathological evaluation

Following dental pulp extirpation, all teeth were fixed in 10% neutral buffered formalin, and samples were decalcified in 20% formic acid solution for ten days. Then, samples were dehydrated in ascending grades of ethanol solutions before being embedded in paraffin blocks. Such blocks were serially sectioned at five μm thickness in a buccolingual direction and stained with hematoxylin and eosin (H&E)^38^. Photomicrographs for stained sections were captured using a digital camera connected with light microscopy (Leica DM100; Leica Microsystems Wetzlar, Germany). Likewise, two blinded authors (D.B.E.F and A.M.Y) independently assessed the dentin bridge formation in the stained histological slides. Furthermore, the dentin bridge thickness was measured using the ImageJ software version 1.53d (NIH, Bethesda, MD, USA) and the digital camera connected with the light microscopy to take photomicrographs of the H&E stained sections, with (40x) being the fixed magnification for the images obtained for analysis.

#### Quantitative real-time polymerase chain reaction (qRT-PCR) evaluation

Total RNA was extracted from dental pulp tissue using QIAmp RNA mini kit (Qiagen, Hilden, Germany) according to the manufacturer's instructions, and their concentration and purity were obtained using a nanodrop (ND-2000) spectrophotometer. cDNA was synthesized using transcriptase reversing (Fermentas, Thermo Fisher Scientific, Waltham, MA, USA). PCR was performed in a total volume of 20 μL using a mixture of 1 μL cDNA, 0.5 mM of each primer (Table 1), and iQ SYBR Green Premix (Bio-Rad 170-880, Bio-Rad Laboratories, Hercules, CA, USA). PCR amplification and analysis were done using Bio-Rad iCycler thermal cycler and the MyiQ real-time PCR detection system. Each assay consists of tested cDNAs in triplicate samples no-template negative control (NTC) was included. Glyceraldehyde 3-phosphate dehydrogenase (GAPDH) served as a reference gene. The relative gene expression of dentin sialophosphoprotein (DSPP), matrix extracellular phosphoglycoprotein (MEPE), and Nestin (NES) were measured by this equation 2^−**ΔΔCT**^^39^.

**Table 1:** Primer sequences of the studied genes.

Target genes	Accession no	Sequence (5' to 3')	Product size
GAPDH (Reference gene)	XM_038448971.1	**F: 5'-** ATGGGCGTGAACCATGAGAA **-3' R: 5'** CAGTGGAAGCAGGGATGATGT**-3'**	238bp
DSPP	XM_038444079.1	**F**: **5**'-CAAACCAGGAGGCAGGAGTAA **-3' R**: **5'-**TGTCTTCAGGGCCATCATCTTC **-3'**	273bp
MEPE	NM_001313825.1	**F**:**5'-** TCTTTTCAGCGTGACTTGGGCA **-3' R**:**5'-** AGGTGCTGGCTCTTGATTTCTTCT **-3'**	247bp
NES	XM_038671838.1	**F: 5' -**GCAGGAGAAGATGCAGACCTAAT **-3' R**: **5-'** GCCTCACTGTCTTCTCTGTTCTT **-3'**	251bp

### Statistical analysis

Data were presented as frequency and percentage values for radiographical evaluation and analyzed using Fisher’s exact test. Cohen’s kappa coefficient was used to assess the inter-rater reliability. For dentin bridge thickness and gene expression analysis, data were reported as mean ± S.E and analyzed using the unpaired *t*-test. The statistical significance was assumed if *p* ≤ 0.05 in all tests. We used SPSS 28 (IBM. Armonk, USA) in performing all statistical analyses.

### Ethical statement

This study was ethically approved by the Institutional Animal Care and Use Committee (IACUC), Cairo University, Egypt (ID: CU III F 29 21). All procedures performed in the study involving animals were in accordance with the National Research Council standards for the care and use of laboratory animals.

## Results

### Radiographic evaluation

**Figure 1:**
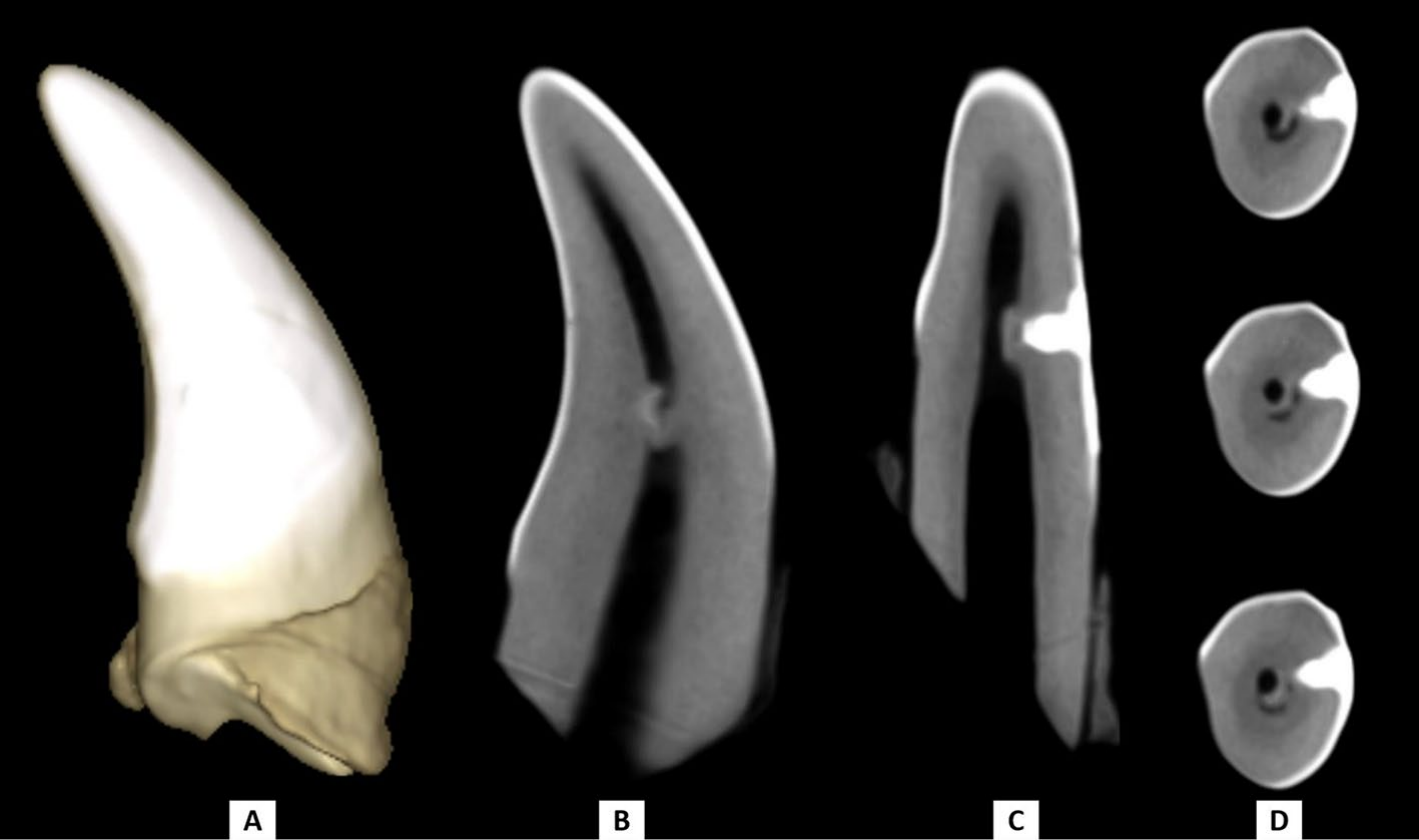
CBCT images of a canine capped with PRP show hard tissue formation between the pulpal space and the cavity treated with pulp capping material; (**A**) 3D image, (**B**) sagittal view, (**C**) coronal view, (**D**) axial views with 0.3 mm inter-slice distance inside the exposure site. This figure was created using the Planmeca Romexis Viewer Launcher software (version 5.3.3.5 R).

**Figure 2:**
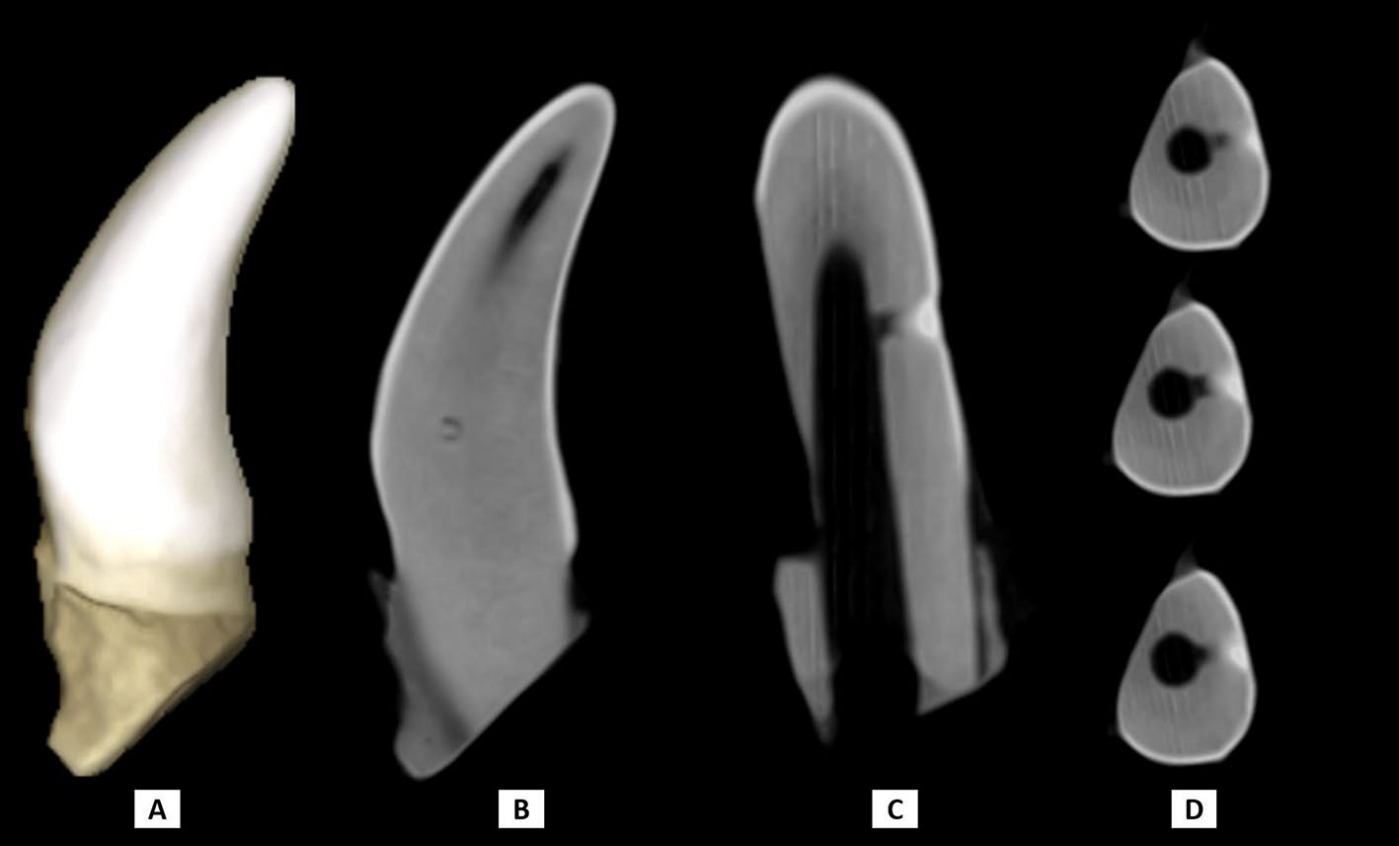
CBCT images of a canine capped with MTA show hard tissue formation between the pulpal space and the cavity treated with pulp capping material; (**A**) 3D image, (**B**) sagittal view, (**C**) coronal view, (**D**) axial views with 0.3 mm inter-slice distance inside the exposure site. This figure was created using the Planmeca Romexis Viewer Launcher software (version 5.3.3.5 R).

Most teeth in both groups showed hard tissue formation; however, there was no statistically significant difference between PRP and MTA (χ2 = 2.27, *p* = 0.440). There was a strong agreement between both evaluators (*k* = 0.899, 95%CI [0.695, 1.102], *p* < 0.001). Details on intergroup comparisons of hard tissue formation categories are summarized in Table 2. (Figs. 1, 2).

**Table 2:** Intergroup comparison of hard tissue formation state.

Hard tissue—*n* (%)	PRP	MTA	χ^2^	*p*-value
No evidence of hard tissue	3 (14.3%)	7 (33.3%)	2.27	0.440
Hard tissue formation	13 (61.9%)	11 (52.4%)		
No restoration found	5 (23.8%)	3 (14.3%)

### Histopathological evaluation

**Figure 3:**
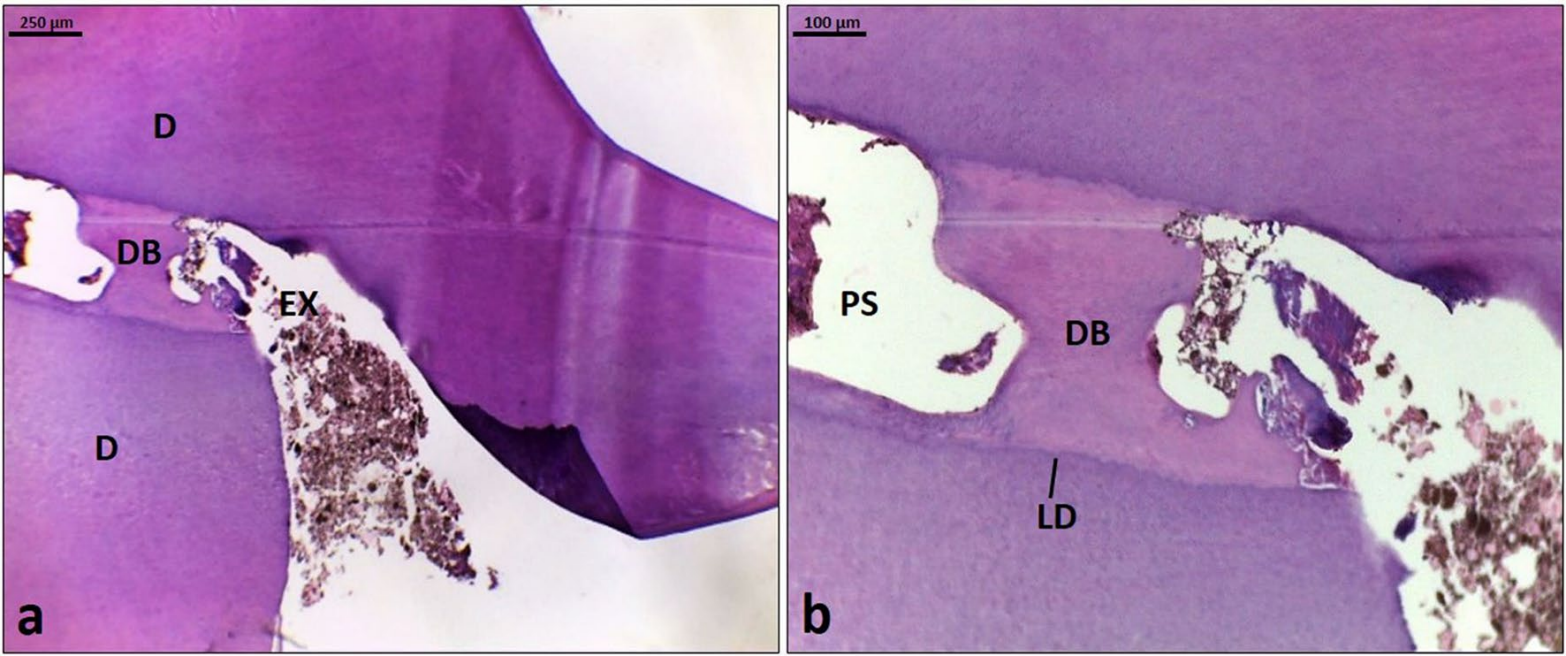
Histologic image (H&E stained) demonstrates tooth capped with PRP. (**a**): primary dentin (D), exposure site (EX), complete reparative dentin bridge (DB) (Scale bar: 250 μm). (**b**) Higher magnification of Fig. 3a shows a homogenous reparative dentin bridge (DB), pulp space (PS), and line of demarcation (LD) (Scale bar: 100 μm).

In the PRP group: The histological sections showed a complete homogenous reparative dentin bridge covering the exposed pulp area and in contact with the lateral dentinal walls (Fig. 3a). There were no cells or blood vessels in such a reparative dentin bridge (Fig. 3b).

**Figure 4:**
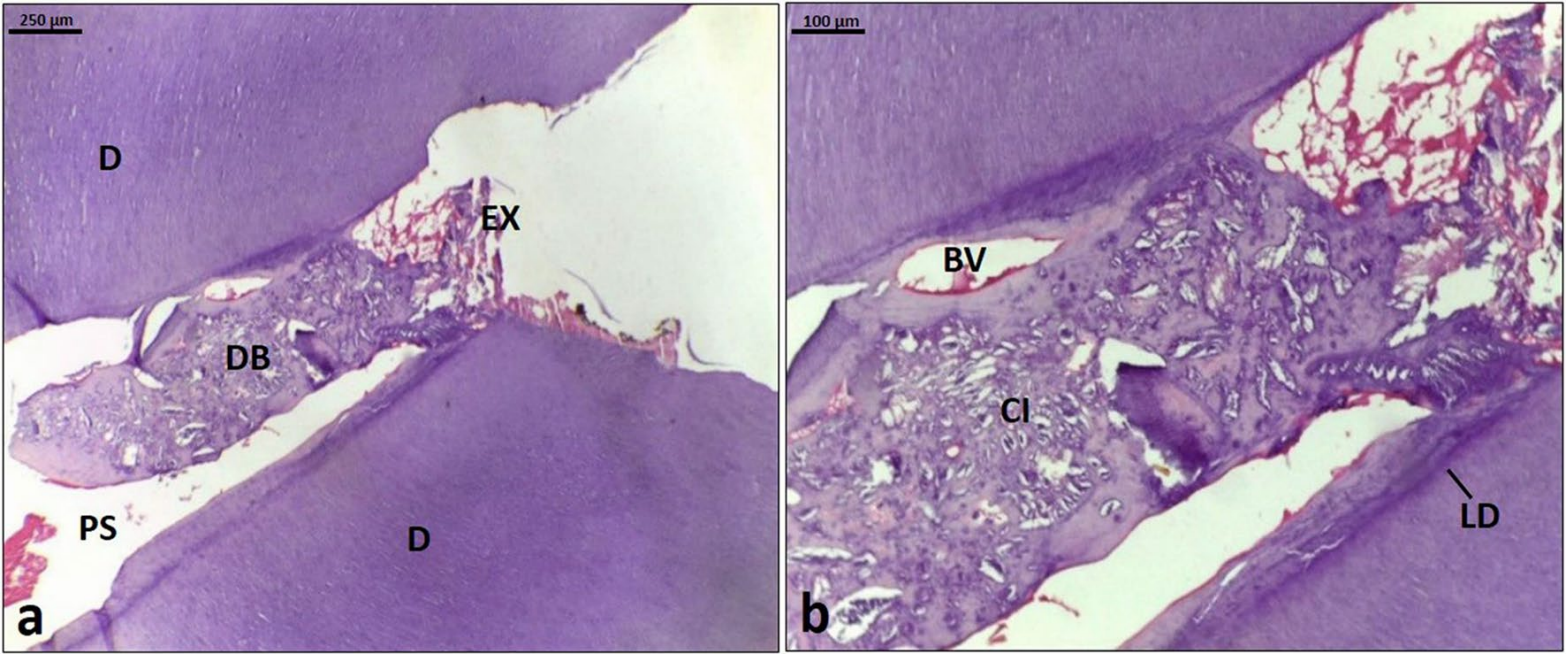
Histologic image (H&E stained) demonstrates tooth capped with MTA. (**a**): primary dentin (D), exposure site (EX), complete reparative dentin bridge (DB), pulp space (PS) (Scale bar: 250 μm). (**b**): A higher magnification of Fig. 4a showing cell inclusions (CI), blood vessel (BV), and line of demarcation (LD) (Scale bar: 100 μm).

In the MTA group: The histological slices showed a complete reparative dentin bridge over the pulp cavity opposite the exposed area and in contact with the lateral dentin walls; however, most of the lateral dentinal walls lost contact with the formed bridge due to its invasion into the intrapulpal space (Fig. 4a). The reparative bridge displayed osteo and vasodentin features and substantial cell and blood vessel inclusions (Fig. 4b).

The quantitative analysis of hard tissue formation revealed that the mean ± S.E of dentin bridge thickness (µm) was in PRP-capped teeth 108.90 ± 2.05 and 110.05 ± 2.62 in MTA-capped teeth, with no statistical significance between the two groups (*p* = 0.732). (Table 3).

**Table 3:** Intergroup comparison of dentin bridge thickness (µm).

Variable	*n*	Mean	Std. err	Std. dev	[95% conf. interval]	*t*-statistic (DF)	*p-value*
PRP	21	108.90	2.05	9.38	[104.64, 113.18]	− 0.34 (40)	0.732
MTA	21	110.05	2.62	11.99	[104.60, 115.51]		
Difference	–	− 1.14	3.32	–	[− 7.86, 5.57]		

### Quantitative real-time polymerase chain reaction (qRT-PCR) evaluation

**Figure 5:**
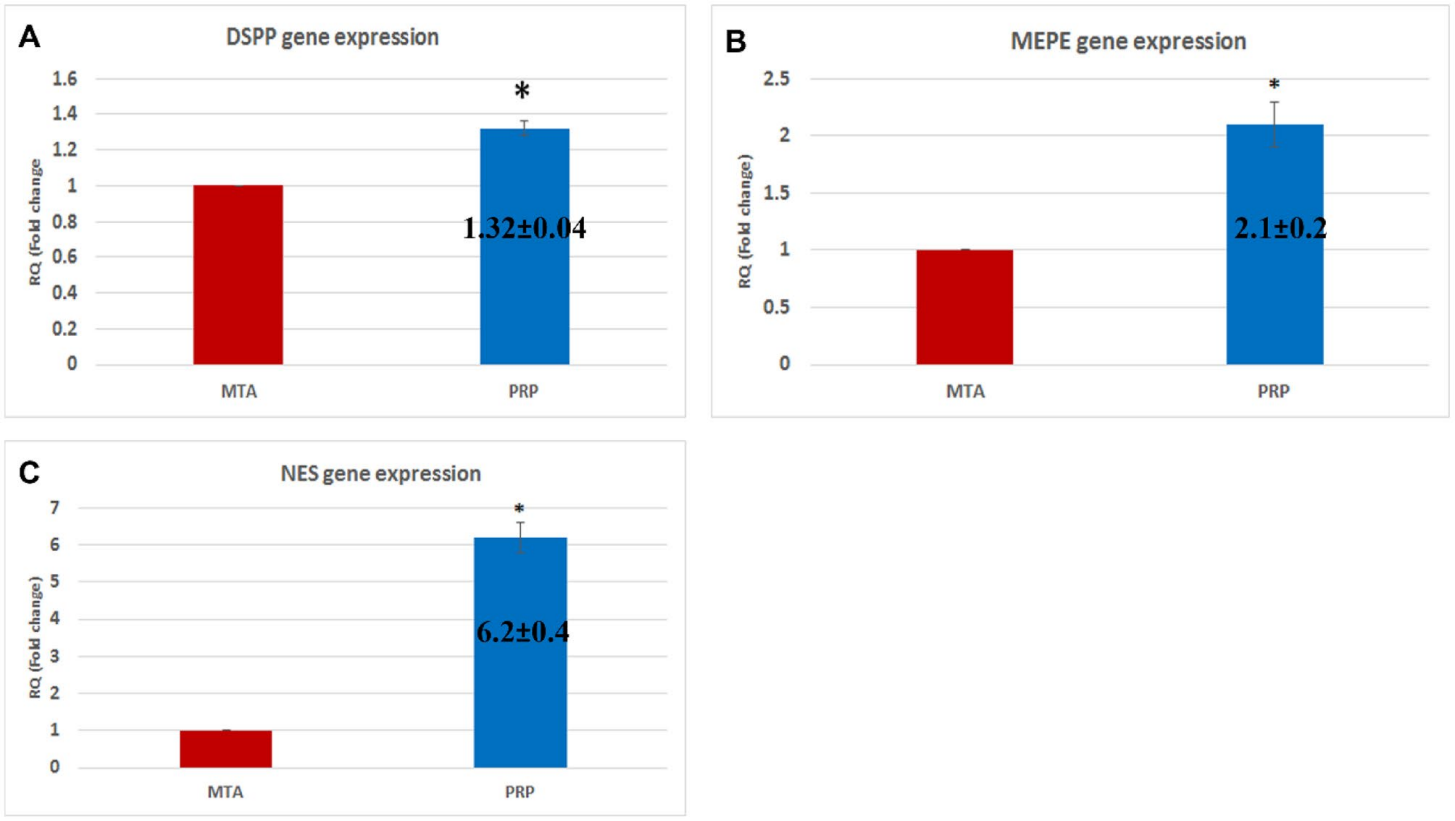
Bar charts summarize mRNA gene expression of (**A**) DSPP, (**B**) MEPE, and (**C**) NES in MTA and PRP groups. Values were expressed as mean ± SEM, *n* = 21/group. Significance at *P* ≤ 0.05. (*) denotes a significant difference in the MTA group.

Compared to the MTA group, mRNA gene expression of DSPP, MEPE, and NES in the PRP group was significantly increased by 1.32, 2.1, and 6.2 folds, respectively (*p* < 0.05). (Fig. 5).

## Discussion

This animal study was designed to investigate the null hypothesis that no radiographic, histopathologic, or dentinogenic gene expression differences exist between PRP and MTA (gold standard) as DPC agents. Our findings revealed that PRP could induce better cellular dentinogenic responses and regenerate dentin with homogenous architecture, rejecting the null hypothesis.

DPC is a practical and conservative approach for preserving tooth vitality without requiring more invasive procedures. The formation of a hard tissue barrier over the pulp's exposure site is a critical indicator in assessing the success of the DPC process^40^. An opaque bridge on radiography is one of the most crucial markers of successful direct pulp capping. Although periapical radiography is commonly used daily to evaluate mineralized tissues, it cannot detect the earliest evidence of dentin formation due to its two-dimensional nature, geometric distortion, and anatomic noise^41,42^. The three-dimensional assessment using cone-beam computed tomography (CBCT), an efficient diagnostic modality in evaluating the dentine bridge developed, makes such detection of freshly formed tertiary dentin more practical and accurate due to its high-resolution images, isotropic volumetric data, multiplanar images, and high sensitivity^43,44^. As such, the clinical absence of pulpitis and the presence of dentin bridge in radiographs indicate that DPC was successful^45,46^. Our radiographic assessment revealed that hard tissue formation was evident in PRP and MTA groups without significant differences, consistent with previous research^29,38,47,48^ except the Shobana et al. clinical trial that revealed a significantly higher volume of the dentine bridge formed by PRP than MTA^49^.

We augmented such CBCT-confirmed biological capability with histopathological evaluation to further investigate dentin bridge formation and structure; thus, our histological assessment also revealed no significant difference in dentin bridge thickness between the PRP-capped and MTA-capped teeth. However, such histological findings showed that the newly formed dentin bridge in the PPR group was homogenous without cellular inclusions; in contrast, that of the MTA formed was osteodentin type (bone-like dentin), which is consistent with previous studies that revealed newly formed calcified hard tissue lacked the histological features of regular dentine after pulp capping with MTA^50–52^. As a result, our findings reasoned that using PRP in DPC induced better cell response than MTA and succeeded in restoring dentin with homogenous architecture rather than dentin bridge with irregular features. In response to pulp exposure, the surviving pulp cells can differentiate into odontoblast-like cells, or the injured odontoblasts start the repair process^53^. Ricucci et al. found that osteodentin formation results from activated pulp cells rather than injured odontoblasts^54^, indicating that PRP's potential to not only induce new odontoblast differentiation but also to reactivate the matrix-secretion of injured odontoblast in the current investigation.

We supported such assumptions with gene expression analyses, as molecular techniques are also well-documented and reliable approaches for evaluating dentin bridge formation^41,52^. Most dentin and bone non-collagenous proteins (NCPs) have been classified as sibling family members with similar structural and molecular characteristics^55^. The sibling family consists of five proteins: Dentin sialophosphoprotein (DSPP), dentin matrix protein 1 (DMP-1), bone sialoprotein (IBSP), osteopontin (SPP1), and matrix extracellular phosphoglycoprotein (MEPE)^56^. The deficiency DSSP, which encodes for dentin sialoprotein (DSP) and dentin phosphoprotein (DPP), causes dentin hypoplasia or dysplasia as well as opalescent, shell-shaped, and malformed deciduous teeth with short roots^57^. The distal-less homeobox 3 (Dlx3), found in odontoblasts, is the first direct target of DSPP, making it a crucial odontoblasts hallmark^58^. MEPE is an extracellular matrix protein found in skeletal and dental tissues. MEPE is present during the development of the craniofacial complex's mineralized tissues (bone, cartilage, and teeth). Concerning the non-mineralized stages of dentinogenesis, osteogenesis, and chondrogenesis, it serves as a particular early matrix-forming signal^56^. Nestin (NES) is an intermediate filament related to neurofilaments found solely in functional odontoblasts, which produce the hard tissue matrix of dentin in young permanent teeth; however, NES is not expressed in older permanent teeth since it has been steadily downregulated^59^. In pathological conditions (e.g., caries and traumatized tooth), its expression is up-regulated in odontoblasts at the injury site, indicating a relationship between tissue repair and nestin up-regulation^60^.

In this light, our findings demonstrated a significant increase in DSPP, mRNA, and NES gene expression in the PRP group compared to the MTA group, indicating that injured odontoblasts maintained secretory activity and re-expressed throughout exposure procedures^61^. Also, MEPE gene expression was significantly upregulated in the PRP group relative to the MTA group. Although both materials induced the reparative dentinogenesis, each had different mechanisms of action since MTA's ability to induce dentinal bridge formation is attributed to the calcium ions released from the material, which react with phosphates in tissue fluid and induce hydroxyapatite formation^62^. Additionally, Aeinehchi et al. found that DPC with MTA could induce cytological and functional alterations in the pulp cells^63^. Laurent et al. revealed that MTA directly affected the dental pulp's regenerative potential and was associated with increased TGF-β1 secretion from pulp cells; such factor directed the progenitor cells' migration to the material-pulp interface and stimulated their differentiation into odontoblastic cells secreting reparative dentin^64^. MTA's high alkalinity also contributes to inflammation and the formation of a hard tissue barrier by providing a favorable environment for cell proliferation, matrix production, and antibacterial activity^65,66^.

On the other hand, the biological impact of PRP depends on the production and release of bioactive molecules, many growth factors, and differentiation factors^67^; such growth factors interact with one another, forming a cascade of various signal proteins that promote the production of proteins by activating gene expression^68^. As a result, it has been proposed that these autologous growth factors are critical to the dentine-pulp complex's repair processes because they alter the odontoblast's ability to proliferate and differentiate^69^.

However, the present research has some limitations; first, this study did not investigate PRP's clinical applicability and limitations regarding its handling, post-operative assessment, setting time, expenses, patient acceptance for injection, and possible inflammatory reactions; hence, our findings should be interpreted with caution and should not be regarded as a clinical recommendation. Second, this study only investigated the effectiveness of PRP as a DPC agent after traumatic exposure, which limits the generalizability of our findings since it differs significantly from carious pulp exposure in terms of pathogenesis and response to treatment. Third, the effectiveness of PRP as a DPC was assessed radiographically, histopathologically, and in terms of dentinogenic gene expression at the transcriptome level without considering the proteomic level. Therefore, further long-term, well-designed clinical research with larger sample sizes in different clinical settings is required to support our findings and further evaluate PRP's clinical applicability and cost-effectiveness as a DPC agent.

## Conclusion

PRP is tissue-compatible and enables mineralized tissue bridge development following DPC similar to MTA (gold standard); still, PRP could generate better cellular dentinogenic responses and restore dentin with homogenous architecture than MTA in dogs' teeth. Moreover, the PRP group showed significantly higher DSPP, MEPE, and NES mRNA gene expression than the MTA group. Given such favorable outcomes and the potential benefits of PRP as an autologous biological agent, PRP might be a promising and effective alternative regenerative agent for DPC. However, further long-term clinical studies with larger sample sizes are required to confirm our conclusions.

## Data Availability

The data that support the findings of this study are available from the corresponding author [A.G.A.K] upon reasonable request.

## Abbreviations

CBCT: Cone-beam computed tomography
DPC: Direct pulp capping
Dlx3: Distal-less homeobox 3
DMP-1: Dentin matrix protein 1
DPP: Dentin phosphoprotein
DSP: Dentin sialoprotein
DSPP: Dentin sialophosphoprotein
IBSP: Bone sialoprotein
MEPE: Matrix extracellular phosphoglycoprotein
MTA: Mineral trioxide aggregate
NES: Nestin
PRP: Platelet-rich plasma
SPP1: Osteopontin
VPT: Vital pulp therapy
